# Shock Index as an indicator for blood transfusion or surgical intervention among multiple trauma patients in Jordan

**DOI:** 10.25122/jml-2024-0348

**Published:** 2025-07

**Authors:** Liqaa Raffee, Khaled Alawneh, Wasfi Al-Salaita, Nour Negresh, Ahmad Al-Omari, Hassan Alawneh, Abeer Khafajah, Majd Elzghairin, Zaid Tashtoush, Yamen Alawneh, Rana Haddad

**Affiliations:** 1 Department of Accident and Emergency Medicine, Faculty of Medicine, Jordan University of Science and Technology, Irbid, Jordan; 2 Department of Diagnostic Radiology and Nuclear Medicine, Faculty of Medicine, Jordan University of Science and Technology, Irbid, Jordan; 3 Department of General Surgery, Jordanian Royal Medical Services, Amman, Jordan; 4 Department of General Surgery, Faculty of Medicine, Al-Balqa Applied University, Al-Salt, Jordan; 5 Department of Medical Engineering, Cardiff University School of Engineering, United Kingdom; 6 Faculty of Medicine, Jordan University of Science and Technology, Irbid, Jordan

**Keywords:** trauma, Shock Index (SI), hemodynamic instability, pneumothorax, blood transfusion, SI, Shock Index, MT, Massive Transfusion, ESPs, Emergency Surgical Procedures, SBP, Systolic Blood Pressure, ER, Emergency Room, INR, International Normalized Ratio, PT, Prothrombin Time, PTT, Partial Thromboplastin Time, rSI, Reverse Shock Index, rSIG, Reverse Shock Index with Glasgow Coma Scale, GCS, Glasgow Coma Scale

## Abstract

Trauma remains a leading cause of mortality worldwide, with uncontrollable bleeding contributing significantly to preventable deaths. This study assessed the utility of the shock index (SI) in predicting clinical outcomes in trauma patients. A retrospective analysis was conducted on 122 trauma patients admitted to King Abdullah University Hospital, Jordan. Patients were categorized into two groups based on their SI: normal (SI < 0.9) and elevated (SI> 0.9). Clinical outcomes, including the need for interventions, blood transfusions, and neurological status, were compared between the groups. Patients with elevated SI had worse neurological outcomes (17% vs. 1.1%, *P* < 0.001), higher rates of airway interventions (23% vs. 4.3%, *P* = 0.005), increased incidence of pneumothorax/hemothorax (*P* = 0.005), and a greater need for blood transfusions (10% vs. 1.1%, *P* = 0.046). Elevated SI was associated with overall hemodynamic instability and worse clinical outcomes, supporting its use as a rapid assessment tool in trauma care. Elevated SI was strongly associated with worse clinical outcomes in trauma patients, including increased need for interventions and higher complication rates. SI proves to be a simple yet effective tool for the rapid assessment of trauma severity, while holding the potential to improve early triage and decision-making within emergency care settings.

## INTRODUCTION

Trauma or physical injury is a significant cause of mortality and disability worldwide [1]. It is a serious global health problem, accounting for approximately one in ten deaths worldwide [2]. Uncontrollable bleeding accounts for 39% of trauma-related deaths and is the leading cause of potentially preventable deaths in patients with major trauma [3]. The Shock Index (SI), commonly referred to as hemodynamic stability, has an accepted range value of 0.5 to 0.7. This index is commonly used to assess the amount of blood loss [3]. A previous study suggested a classification system that stratifies patients into four categories based on SI on hospital arrival: *no shock* (< 0.6), *mild shock* (0.6–1.0), *moderate shock* (1.0–1.40), and *severe shock* (≥ 1.4). Patients with ‘moderate shock’ and ‘severe shock’ received massive transfusion (MT)(≥10/24 h) at proportions of 31% and 57%, respectively [4].

In both civilian and military populations, SI has demonstrated strong predictive value for outcomes such as massive transfusion and emergency surgical procedures, particularly in resource-limited or austere environments [5]. While systolic blood pressure (SBP) and SI are commonly used to predict outcomes in trauma patients, evidence suggests that the optimal threshold values for these indicators can vary depending on the underlying cause of trauma and the patient’s clinical condition [6–10]. Factors such as advanced age, pre-existing hypertension, and the use of β-blockers or calcium channel blockers have been shown to diminish the predictive strength of SI for 30-day mortality [6]. Consequently, the effectiveness of SBP and SI as evaluation tools in older trauma patients or those receiving antihypertensive therapy remains inadequately established [11].

## MATERIAL AND METHODS

### Study design and setting

This was a retrospective cross-sectional study conducted at King Abdullah University Hospital (KAUH) in Irbid, Jordan. Data were collected for all patients admitted to the Emergency Room (ER) between March 2016 and March 2023. Patients under the age of 18 and pregnant women were excluded from the study.

### Questionnaire and data collection

**Figure 1:**
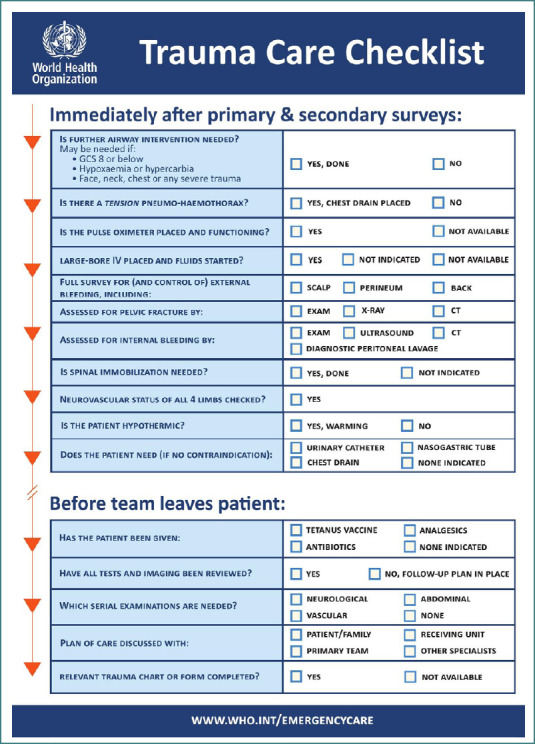
WHO Trauma Care Checklist

Data were extracted using a structured spreadsheet based on the World Health Organization (WHO) Trauma Care Checklist (Figure 1) [12]. Variables in the data collection spreadsheet included socio-demographic characteristics, comorbidities, clinical manifestations, and imaging findings. Information was gathered by reviewing each patient’s history, physical examination notes, and radiological reports available in the EMRs.

### Statistical analysis

For continuous variables, the median and interquartile range (IQR) were used to describe central tendency and deviation. Categorical variables were summarized using frequencies and percentages (%). The association between demographic, clinical, and laboratory variables with study groups was assessed using the Wilcoxon (Mann–Whitney U test) test for continuous variables, while the chi-squared (χ^2) and Fisher's exact tests were employed for categorical variables, particularly when the count within a category was less than 5. Statistical significance was set at a *P* value of < 0.05. All statistical analyses were performed using SPSS version 26.

## RESULTS

A total of 122 patients were included in the study. Among them, 30 patients (24.6%) had an elevated SI (SI> 0.9), and 92 patients (75.4%) had a normal SI (SI ≤ 0.9). Most patients were men (74%), with a median age of 33 years. Most patients (93%) presented with a Glasgow Coma Scale (GCS) score of 8 or higher, and 11 patients (9%) required airway intervention. The median heart rate was 84 beats per minute, the median respiratory rate was 18, and the median temperature was 36.9 degrees Celsius. Additionally, 11 patients (9%) experienced tension pneumothorax or hemothorax (Table 1).

**Table 1:** Comparison of demographic and clinical indicators by shock index in trauma patients

Characteristic	Elevated *n* = 30^1^	Normal *n* = 92^1^	*P* value ^2^	Overall *n* = 122^1^
**Gender**			0.6	
Female	9 (30%)	23 (25%)		32 (26%)
Male	21 (70%)	69 (75%)		90 (74%)
**Age**	36 (13, 40)	32 (25, 43)	0.3	33 (22, 43)
**Glasgow coma scale**			0.007	
8 or more	24 (80%)	89 (97%)		113 (93%)
Less than 8	6 (20%)	3 (3.3%)		9 (7.4%)
**Was further airway intervention needed?**	7 (23%)	4 (4.3%)	0.005	11 (9.0%)
**Heart rate**	94 (81, 119)	80 (75, 90)	<0.001	84 (78, 95)
**Respiratory rate**	19 (18, 22)	17 (16, 18)	<0.001	18 (16, 19)
**Temperature**	36.9 (36.8, 37.0)	36.8 (36.7, 37.0)	0.2	36.9 (36.7, 37.0)
**Tension pneumothorax/hemothorax**	7 (23%)	4 (4.3%)	0.005	11 (9.0%)

^1^ *n* (%); Median (IQR)

^2^ Pearson’s Chi-squared test; Wilcoxon rank sum test; Fisher’s exact test

Patients in the elevated SI group were less likely to have a GCS ≥ 8 compared to those with a normal SI (80% vs. 97%, *P* = 0.007). Also, patients with an elevated SI had a higher need for airway intervention (23% vs. 4.3%, *P* = 0.005). Patients in the elevated SI group experienced a higher heart rate (*P* < 0.001), higher respiratory rate (*P* < 0.001), and higher rates of tension pneumothorax/hemothorax (*P* = 0.005) when compared to the normal SI group. No significant differences were observed in gender, age, or temperature between the two groups.

Eight patients presented with external bleeding, while 76% had intact neurovascular examinations. Only four patients (3.3%) required a blood transfusion. The median ICU stay was 3.5 days, whereas the median hospital stay length was 4 days. The median Injury Severity Score (ISS) was 8. All patients passed away in the hospital (Table 2).

**Table 2:** Patient outcomes compared by shock index score categories

Characteristic	Elevated, *n* = 30^1^	Normal, *n* = 92^1^	*P* value^2^	Overall, *n* = 122^1^
**External bleeding**	0 (0%)	8 (8.7%)	0.2	8 (6.6%)
**Neurovascular exam**			<0.001	
Drowsy	0 (0%)	7 (7.9%)		7 (5.9%)
Intact	21 (70%)	70 (79%)		91 (76%)
Intubated	4 (13%)	0 (0%)		4 (3.4%)
Loss of consciousness	5 (17%)	1 (1.1%)		6 (5.0%)
Normal	0 (0%)	11 (12%)		11 (9.2%)
**Need for blood transfusion**	3 (10%)	1 (1.1%)	0.046	4 (3.3%)
**INR**	1.00 (1.00, 1.10)	1.10 (1.02, 1.20)	0.10	1.09 (1.00, 1.20)
**PT**	14.00 (13.20, 14.60)	14.30 (13.55, 15.75)	0.4	14.15 (13.30, 15.63)
**PTT**	27.00 (26.00, 29.00)	26.40 (24.60, 28.45)	0.2	26.55 (25.00, 28.73)
**PLT**	281 (249, 299)	241 (198, 287)	0.013	255 (204, 293)
**ICU length stay**	4.0 (1.0, 8.0)	3.0 (1.0, 5.0)	0.5	3.5 (1.0, 5.0)
**Hospital length stay**	8 (2, 21)	4 (2, 7)	0.2	4 (2, 7)
**Injury severity score**	6 (3, 23)	8 (4, 16)	0.8	8 (4, 16)
**Mortality after ER**	30 (100%)	92 (100%)		122 (100%)

^1^ *n* (%); Median (IQR)

^2^ Fisher’s exact test; Wilcoxon rank sum test

In terms of neurologic findings, loss of consciousness was significantly more common in the elevated SI group (17% vs. 1.1%, *P < 0.001*). Additionally, blood transfusion was more frequently required in this group (10% vs. 1.1%, *P = 0.046*). Patients with elevated SI also had higher platelet counts, with this difference reaching statistical significance (*P = 0.013*). No significant differences were observed between groups in terms of external bleeding, international normalized ratio (INR), prothrombin time (PT), partial thromboplastin time (PTT), ICU length of stay, total duration of hospitalization, or ISS. Based on SI classification by shock severity, 24 patients (19%) were categorized as having no shock, 72 patients (59%) had mild shock, and 26 patients (21%) experienced moderate to severe shock. The results were consistent with the prior classification (Tables 3 and 4).

**Table 3:** Baseline characteristics and clinical findings stratified by shock index (SI) category

Characteristic	No shock *n* = 24^1^	Mild shock *n* = 72^1^	Moderate/Severe shock *n* = 26^1^	*P* value^2^	Overall *n* = 122^1^
**Gender**				0.5	
Female	5 (21%)	18 (25%)	9 (35%)		32 (26%)
Male	19 (79%)	54 (75%)	17 (65%)		90 (74%)
**Age**	36 (27, 48)	31 (23, 41)	36 (21, 40)	0.3	33 (22, 43)
**Glasgow coma scale**				0.006	
8 or more	24 (100%)	69 (96%)	20 (77%)		113 (93%)
Less than 8	0 (0%)	3 (4.2%)	6 (23%)		9 (7.4%)
**Was further airway intervention needed?**	1 (4.2%)	3 (4.2%)	7 (27%)	0.004	11 (9.0%)
**Heart rate**	71 (67, 77)	87 (80, 94)	85 (81, 121)	<0.001	84 (78, 95)
**Respiratory rate**	16.5 (16, 17)	17.5 (16, 19)	18 (18, 22)	<0.001	18 (16, 19)
**Temperature**	36.80 (36.7, 36.9)	36.8 (36.7, 37.0)	36. (36.9, 37.0)	0.3	36.9 (36.7, 37.0)
**Tension pneumothorax/hemothorax**	1 (4.2%)	4 (5.6%)	6 (23%)	0.024	11 (9.0%)

^1^ *n* (%); Median (IQR)

^2^ Pearson’s Chi-squared test; Kruskal-Wallis rank sum test; Fisher’s exact test

**Table 4:** Patient outcomes compared by shock index score categories

Characteristic	Mild shock *n* = 72^1^	No shock *n* = 24^1^	Moderate/Severe shock *n* = 26^1^	*P* value^2^	Overall *n* = 122^1^
**External bleeding**	5 (6.9%)	3 (13%)	0 (0%)	0.2	8 (6.6%)
**Neurovascular exam**				<0.001	
Drowsy	7 (10%)	0 (0%)	0 (0%)		7 (5.9%)
Intact	51 (74%)	23 (96%)	17 (65%)		91 (76%)
Intubated	0 (0%)	0 (0%)	4 (15%)		4 (3.4%)
Loc	1 (1.4%)	0 (0%)	5 (19%)		6 (5.0%)
Normal	10 (14%)	1 (4.2%)	0 (0%)		11 (9.2%)
**Need for blood transfusion**	1 (1.4%)	0 (0%)	3 (12%)	0.064	4 (3.3%)
**INR**	1.12 (1.03, 1.20)	1.05 (0.96, 1.15)	1.00 (1.00, 1.09)	0.053	1.09 (1.00, 1.20)
**PT**	14.60 (13.60, 15.75)	13.90 (12.55, 14.65)	14.00 (13.05, 14.83)	0.2	14.15 (13.30, 15.63)
**PTT**	26.40 (24.60, 28.40)	26.70 (24.70, 28.55)	27.50 (26.00, 29.00)	0.4	26.55 (25.00, 28.73)
**PLT**	251 (204, 297)	223 (179, 273)	281 (255, 299)	0.026	255 (204, 293)
**ICU length of stay**	3.0 (1.0, 5.0)	3.0 (1.3, 4.0)	4.5 (2.5, 11.0)	0.5	3.5 (1.0, 5.0)
**Hospital length of stay**	4 (2, 7)	4 (3, 6)	8 (2, 21)	0.4	4 (2, 7)
**Injury severity score**	6 (4, 12)	9 (4, 16)	7 (2, 26)	0.6	8 (4, 16)
**Mortality after ER**	72 (100%)	24 (100%)	26 (100%)		122 (100%)

^1^ *n* (%); Median (IQR)

^2^ Fisher’s exact test; Kruskal-Wallis rank sum test

## DISCUSSION

Trauma and physical injury are major contributors to mortality and disability globally, posing a significant public health challenge and accounting for about 10% of deaths worldwide. Uncontrolled bleeding is responsible for 39% of trauma-related deaths and is the leading cause of potentially preventable fatalities in patients with severe trauma [1,2]. Advanced age, hypertension, and the use of β-blockers or calcium channel blockers can impact the association between SI and 30-day mortality. The effectiveness of SBP and SI as assessment tools in geriatric trauma patients or those on antihypertensive medications remains unclear. Therefore, this study aimed to investigate the utility of SI as a potential indicator to guide clinical management decisions. Specifically, the need for blood transfusions and other critical interventions was evaluated in a cohort of patients with multiple traumas admitted to a tertiary care center in Jordan.

In our cohort, a marked elevation in the shock index (SI > 0.9) was observed, and the GCS was significantly higher in those with elevated SI compared to those with lower SI, in addition to a significantly higher need for airway intervention. Several studies utilized the reverse shock index (rSI), a ratio of systolic blood pressure and heart rate multiplied by the GCS (rSIG), with the intent of forming a prediction for in-hospital trauma patients’ mortality. The rSI was introduced by a research group in Taiwan in response to the observation that, in many trauma cases, systolic blood pressure tends to decrease more substantially than heart rate. This dynamic often results in an rSI value less than 1, which has been associated with worse outcomes and increased mortality risk [7,13].

Furthermore, our results revealed that higher SI was also associated with higher pulse rates, respiratory rates, and increased incidence of tension pneumothorax or hemothorax, which are critical factors contributing to hemodynamic instability in trauma patients. A study by Kheirbek *et al*. investigated the association between prehospital SI and the presence of significant injuries, which included pneumothorax and hemothorax, and found that patients with an elevated prehospital SI had significantly higher rates of injuries, need for transfusion, and death at the emergency department [14]. However, prehospital hypotension alone was not a significant indicator of significant injuries or the need for intervention and showed worse performance than SI [14]. Surprisingly, the association between SI and significant injury was higher in patients with normal blood pressure compared to those with prehospital hypotension, suggesting the need to increase the threshold for defining hypotension to 105 or 110 mmHg, as in several studies [14-16]. These findings highlight a potential issue where some patients with normal blood pressure might not be adequately triaged with full trauma team activation, despite having significant injuries that could have been detected through the use of SI [17,18].

This study illustrated several strengths, in which an initial focus was placed on a critical global health issue, which is trauma-related mortality and disability, by exploring the use of the shock index as a simple, reproducible, and efficient tool for assessing trauma patients. Additionally, we utilized real-world data from King Abdullah University Hospital over a seven-year period in Jordan, which provides valuable insights into patient care in a practical clinical setting, particularly in the Middle East, where data on SI usage is limited. The single-center, retrospective design inherently introduces selection bias and may limit the generalizability of our findings to other trauma care settings. Retrospective data collection relies on the accuracy and completeness of medical records, which could introduce information bias and affect data reliability. Additionally, the inability to control confounding variables, such as pre-existing comorbidities, medication use, and differences in trauma care protocols, may have influenced the outcomes observed. Additionally, the study's internal and external validity may be affected by the limited sample size, particularly the small number of patients (*n* = 30) exhibiting an elevated SI >0.9, which could impact the robustness and generalizability of the findings.

The generalizability of our findings may be limited due to the exclusion of patients under 18 years of age and pregnant women. As such, the results should be interpreted with caution and regarded as preliminary. Future multicenter prospective studies are warranted to validate these findings, address current limitations, and provide more definitive evidence on the clinical utility of the SI in trauma care. Future research should also focus on including diverse patient groups to broaden applicability and investigate the utility of SI in patients with specific health conditions. Comparative studies could assess SI against other existing tools, and implementation research should explore how SI can be integrated into trauma care protocols in various clinical settings, especially those with limited resources, to improve clinical outcomes and decision-making processes.

## CONCLUSION

Our findings suggest that SI may serve as a useful tool for assessing trauma severity and predicting clinical outcomes in emergency patients. Elevated SI was associated with worse neurological outcomes, increased need for interventions and transfusions, and higher rates of significant injuries. However, given the study’s single-center, retrospective design, limited sample size, and potential biases, these results should be interpreted with caution. Our study serves as a preliminary investigation, and future prospective, multicenter research is needed to validate these findings, improve the robustness of conclusions, and explore the broader applicability of SI in diverse trauma populations.

## Data Availability

The datasets used and/or analyzed during the current study are available from the corresponding author upon reasonable request.
